# Effect of Pyridine on the Mesophase of Teraryl Liquid Crystals: A New Series of Nematic Liquid Crystals Named 2-(4-Alkoxybiphen-4′-yl)-5-methylpyridines

**DOI:** 10.3390/ijms17030344

**Published:** 2016-03-07

**Authors:** Win-Long Chia, Yu-Sin Huang

**Affiliations:** Department of Chemistry, Fu Jen Catholic University, New Taipei City 24205, Taiwan; hu26419@hotmail.com

**Keywords:** liquid crystals, teraryl mesogen, picoline terminus, nematic phase

## Abstract

A new series of teraryl 2-(4-alkoxybiphen-4′-yl)-5-methylpyridines (*n*O-PPPyMe, *n* = 3–8) nematic liquid crystal compounds, bearing a biphenylene core and a picoline terminus, were synthesized using a short two-step reaction, and overall yields between 34% and 38% were obtained. Spectral analysis results were in accordance with the expected structures. The thermotropic behavior of the teraryl liquid crystal compounds was investigated through polarized optical microscopy and differential scanning calorimetry. All compounds exhibited a solely enantiotropic nematic phase at the medium–high temperature range of 162.4–234.2 °C. Furthermore, the results for the *n*O-PPPyMe series were analyzed relative to three other compound series, *m*O-PPPyCN (*m* = 2–8), *i*O-PPQMe (*i* = 3–8) and *x*O-PPyPMe (*x* = 1–10). Consequently, the effect of pyridine on the mesophase of teraryl liquid crystals was demonstrated.

## 1. Introduction

In liquid crystals (LCs), when the structure of a molecule is changed, many molecular parameters are affected, which may differentially influence the properties of the LCs. Thus, the relationships between the molecular structure of LC and its properties must be clarified through systematic studies of the effects of molecular structural change. Although the molecular structures of thermotropic mesogens have a common geometrical (*i.e.*, rod- or lath-like) feature, the exact factors favoring the formation of a nematic phase and/or only the nematic phase with a low melting point and high nematic stability remain unknown [1,2].

Pyridine is related to benzene in that one of the CH groups of benzene is replaced by a nitrogen atom. The resonance stabilization energy of pyridine (117 kJ·mol^−1^) is slightly lesser than its benzene analog (150 kJ·mol^−1^). Geometrically, pyridine is a planar molecule, having a structure of a slightly distorted hexagon because the C–N bonds (134.0 pm) are shorter than the C–C bonds (139.5 pm) [3]. Furthermore, pyridine has a dipole moment of 2.19 debyes, which is approximately 50% of that of a cyano group (approximately 4.0 debyes) [4]. Investigating how a mesomorphic phase is changed when pyridine is used as a moiety in the mesogenic core of a LC molecule is expected to yield interesting results.

Because of synthetic difficulties, only a few studies investigated the thermotropic trends within a homologous series of pyridine-containing LC compounds [1,5,6,7]. Synthesis of pyridine-containing LCs generally involves a cyclisation reaction using an enamine to react with a vinyl ketone to obtain a dihydropyran derivative, which was then used to react with hydroxylamine hydrochloride to form pyridine [6]. Another method in preparing 2,5-disubstituted pyridines is using the reaction of acetophenone with ethyl formate in the presence of sodium, followed by cyclisation with cyanoacetamide, substitution of oxygen by chlorine, and reductive elimination of chlorine [8,9,10].

Pyridine-containing LC compounds were also prepared by other methods [11,12,13,14,15,16], such as by cross coupling of arylboronic acids with halopyridines in the presence of a palladium complex [11,12,13], and by reacting 4-*n*-alkoxybenzyl amine with 2,2-dichloro-1-(4-methylphenyl)cyclopropane carbaldehyde at an elevated temperature [14,15,16]. Recent progress in organometallic coupling methods using trifluoroborate derivatives reported by Molander, *et al.* [17,18] has been particularly successful in the coupling of heterocyclic moieties. Seed, *et al.* [19] modified this method to prepare a new class of mesogenic materials that exhibit the smectic C phase. Although these methods are valuable in preparing heterocyclic LC molecules, most methods have their limitations, requiring a relatively expensive catalyst, and occasionally requiring many low-yields synthetic steps. Thus, a facile and inexpensive synthetic method is required to investigate the influence of pyridine moieties on the physical properties of LC materials.

We previously reported a novel method for synthesizing pyridine-containing LC compounds [20,21,22,23,24,25]. Here, we report a convenient, short two-step synthesis method of a homologous series of 2-(4-alkoxybiphen-4′-yl)-5-methylpyridines (*n*O-PPPyMe; *n* = 3–8; propyloxy to octoxy). In addition, we compared the length of the alkoxy chain with the thermotropic behavior of this teraryl mesogenic homologous series. The targeted molecules contain an alkoxy tail, a biphenyl moiety, and a picoline terminus.

## 2. Results and Discussion

### 2.1. Synthesis

**Scheme 1:**
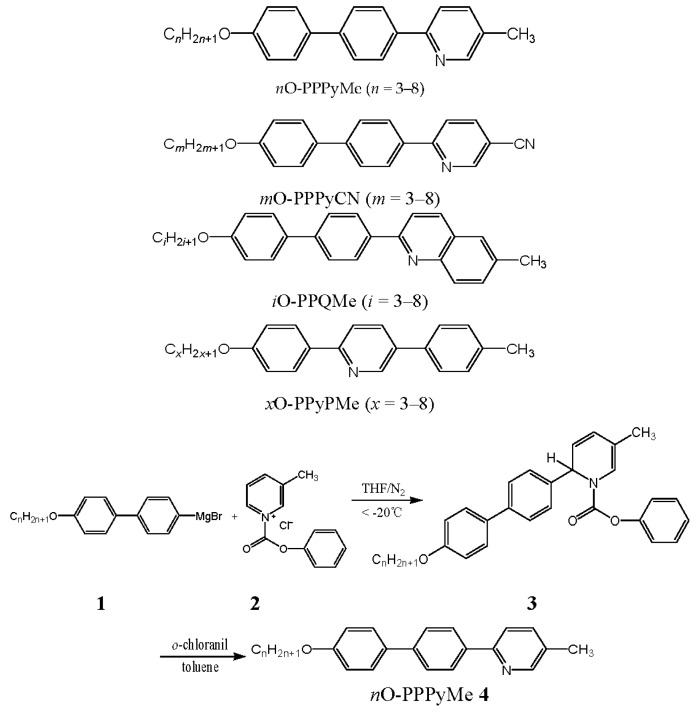
Synthetic route for synthesizing *n*O-PPPyMe.

A new homologous series of *n*O-PPPyMe (*n* = 3–8) was obtained first through the regioselective addition of Grignard reagents to activated 1-acyl-3-picolinium salts to preferentially form 1,2-dihydro-5-picolines, which were subsequently aromatized through a mild oxidation reaction (Scheme 1).

Grignard reagent of **1** was prepared by reacting magnesium with the appropriate 4′-alkoxy-4-bromobiphenylene, which was obtained by reacting 4′-hydroxy-4-bromobiphenylene with an appropriate bromoalkane. The reaction between 3-picoline and phenyl chloroformate produced 3-picolinium chloride **2**. The reaction between Grignard reagents **1** and 3-picolinium chloride **2** generated the expected 1,2-dihydro-5-picoline adduct **3**. Adduct **3** was then oxidized using *o*-chloranil to produce the desired compound **4** (*n*O-PPPyMe, *n* = 3–8).

The strong electrophile, phenyl chloroformate, was used to enhance the reactivity of picoline and aid the nucleophilic attack of the Grignard reagent. This synthetic methodology, according to the HSAB rule, favored Grignard nucleophilic α-regioselectivity at the picoline ring [26,27]. Because of the high polarity difference between the major α and minor γ products, trace amounts of γ products were easily separated through liquid chromatography using an eluant system of 2:1 methylene chloride:hexane. The yields of this two-step reaction ranged from 34% to 38% (Table 1). Analytically pure products were collected by recrystallizing them several times with methylene chloride and ethyl acetate.

### 2.2. Thermotropic Studies

Phase transition temperatures and the associated enthalpy changes in *n*O-PPPyMe compound series were determined through differential scanning calorimetry (DSC). The heating and cooling rates were set at 5 °C·min^−1^. The corresponding mesophases of the *n*O-PPPyMe compounds were identified by observing their textures through polarized optical microscopy, as shown in Table 2 [28].

With picoline as the terminus, an enantiotropic nematic phase was the sole mesophase observed in all *n*O-PPPyMe (*n* = 3–8) compounds. The nematic phase was observed at the medium–high temperature range of 165.7–232.9 °C. Regarding the thermal stability of the *n*O-PPPyMe (*n* = 3–8) compounds, 5% of the initial mass lost was at 260–291 °C.

**Figure 1:**
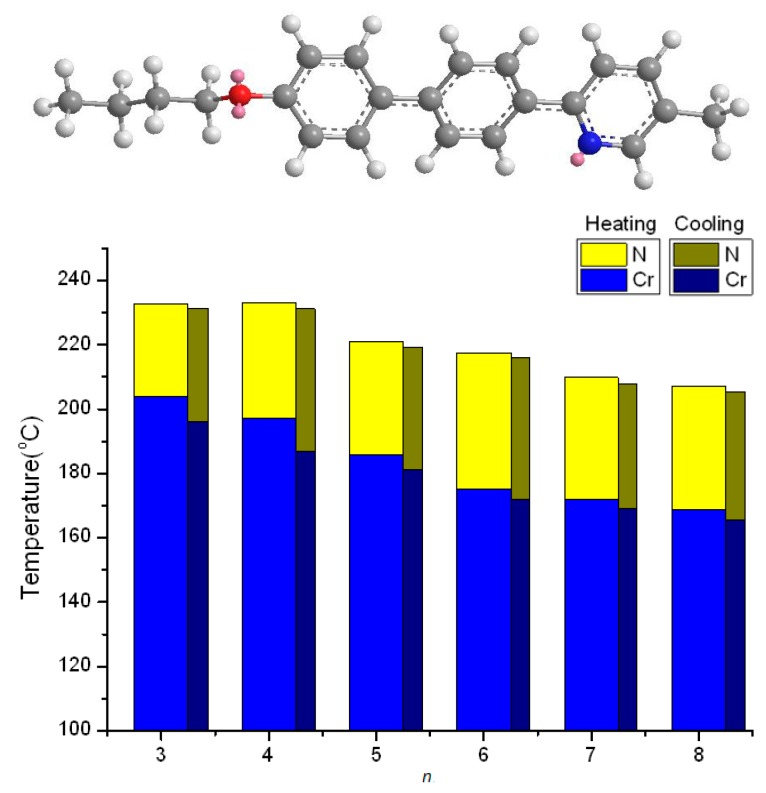
Molecular geometry of *4*O-PPPyMe (calculated by MM2) and the plot of transition temperatures of heating and cooling cycles as a function of the terminal alkoxy chain of the *n*O-PPPyMe (*n* = 3–8) homologs.

Figure 1 is a plot of the transition temperatures of the heating and cooling cycles against the number of carbons in the terminal alkoxy chain of the *n*O-PPPyMe (*n* = 3–8) compounds. The melting point reduced steadily from 203.8 to 168.9 °C in a narrow range (difference: 34.9 °C), with an increase in the length of the alkoxy chain; a similar decreasing trend was observed for the freezing point from 196.0 to 165.7 °C (difference: 30.3 °C). Slightly hysteresis (7.8, 10.1, 4.8, 3.2, 3.1, and 3.2 °C for *n* = 3–8, respectively) was observed in all *n*O-PPPyMe (*n* = 3–8) compounds. The degree of hysteresis showed alternating changes when *n* = 3–5, and stabilized to a narrow range of 3.1 to 3.2 °C when *n* = 6–8. We believe that the geometrically asymmetric picoline terminus in *n*O-PPPyMe plays a major role in hindering nucleation during crystallization from the mesophase and delaying the freezing process. The influence of the asymmetric picoline terminus on hysteresis was substantial when alkoxy chains were short and even, and was somewhat obscured when alkoxy chains were long. The highest degree of hysteresis, 10.1 °C, was found when *n* = 4.

Consequently, nematic phase lengths at cooling (35.2, 44.3, 38.0, 44.2, 39.0, and 39.5 °C for *n* = 3–8, respectively) were slightly longer than those at heating (29.0, 35.9, 35.0, 42.6, 37.8, and 38.0 °C for *n* = 3–8, respectively). The differences of the nematic phase lengths between heating and cooling cycles (6.2, 8.4, 3.0, 1.6, 1.2, and 1.5 °C for *n* = 3–8, respectively) corresponded to the hysteresis phenomena of *n*O-PPPyMe compounds.

Typically, the anisotropy of molecular polarizability is higher for alkoxy chains with an even number of carbons, and their nematic-to-isotropic transition temperatures (*T_NI_*) are higher than those of alkoxy chains with an odd number of carbons. Thus, *T_NI_* varies in a zigzag manner with *n*, and an odd–even effect is observed [29]. Furthermore, a progressively decreasing trend in the anisotropy of the molecular polarizability of the longer alkoxy homologs was generally observed in the nematic phase. Damping of the stepwise decline in *T_NI_* can be explained by the statistical increase in the numbers of possible non-extended conformations [29].

In this study, a pseudo step-wise decrease was apparent if the *T_NI_* for *n* = 3–8 were grouped into three pairs (*n* = 3 and 4; 5 and 6; and 7 and 8). The *T_NI_* for each pair differed only slightly from each other (232.8 and 232.9 °C, 220.9 and 217.7 °C, and 209.9 and 206.9 °C for *n* = 3 and 4, 5 and 6, and 7 and 8, respectively, for heating); similar results were observed during cooling. Mild damping in the stepwise decrease of *T_NI_* was observed. The mildness of damping can be ascribed to the existence of a slightly bending picoline terminus in *n*O-PPPyMe, which created more free volume (void) for the motion of alkoxy chains in the fluid nematic phase.

An alternating change in the nematic phase lengths was observed for *n* = 3–8 homologs. The nematic phase lengths for the heating cycle were 35.9, 42.6, and 38.0 °C for *n* = 4, 6, and 8, and 29.0, 35.0, and 37.8 °C for *n* = 3, 5, and 7, respectively. Similar trends were observed during cooling (44.3, 44.2, and 39.5 °C for *n* = 4, 6, and 8, and 35.2, 38.0, and 39.0 °C for *n* = 3, 5, and 7, respectively). The alternating change in the nematic phase lengths was caused by the greater anisotropy of molecular polarizability (thus raising the *T_NI_*) and the greater influence of the asymmetric mesogenic core on reducing melting and freezing temperatures for the alkoxy chains with an even number of carbons. The longest nematic phase range was 44.3 °C of 4*O*-PPPyMe at cooling.

**Figure 2:**
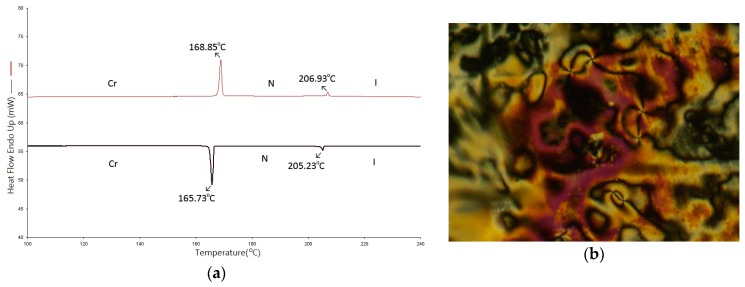
*8*O-PPPyMe: (**a**) thermograms of a second DSC scan at a heating (**red**) and cooling (**black**) rate of 5 °C·min^−1^; and (**b**) polarized optical micrograph originating from the isotropic phase on cooling to 204.8 °C, nematic schlieren texture, ×200.

Figure 2a,b depicts the DSC thermograms and the polarized optical micrograph of the nematic schlieren texture of *8*O-PPPyMe, respectively. During the second scan of the heating process, two endothermic peaks were observed at 168.9 and 206.9 °C, and, during the cooling process, two exothermic peaks were observed at 205.2 and 165.7 °C. The enantiotropic nematic phase and the small cusps of the nematic-to-isotropic transition, *T_NI_* (or *T_IN_*), of *8*O-PPPyMe were easily observed in the thermograms. The typical nematic schlieren texture could be identified by the appearance of characteristic bright colorful brushes and a dark area, in which the n-director of liquid crystal molecules is located parallel or normal to the polarizer with a planar alignment.

Although the molecular weights of *n*O-PPPyMe (*n* = 3–8) compounds were not high (303.4–373.5 g·mol^−1^), relatively high enthalpies of nematic-to-isotropic transitions, H_NI_ (or H_IN_), of 0.55–1.00 kJ·mol^−1^ were found. If these enthalpies are calculated and expressed in terms of entropy changes and are scaled by the gas constant, S_NI_/R is 1.56, 1.54, 1.92, 1.37, 1.21, and 1.67 kJ·mol^−1^ for *n* = 3–8, respectively [30,31,32,33]. High enthalpies H_NI_ and entropies S_NI_/R indicate relatively strong intermolecular attractive forces in the fluid nematic phase.

**Figure 3:**
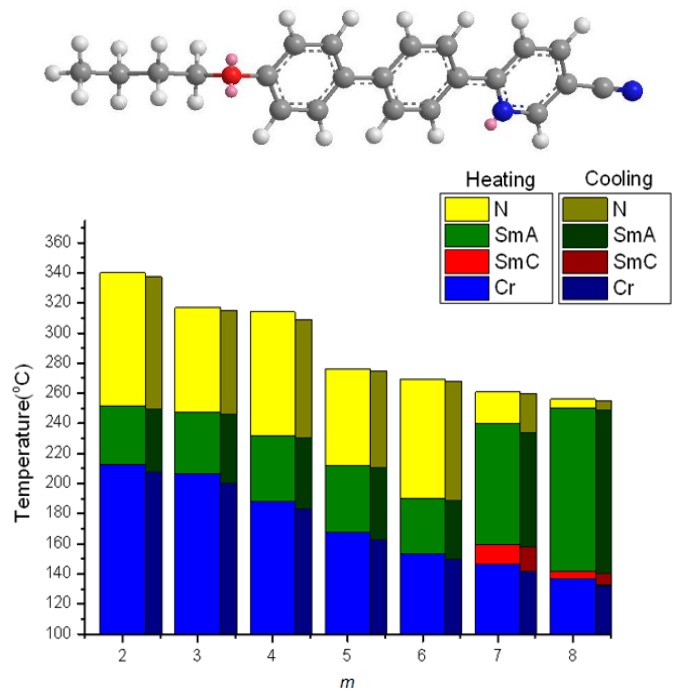
Molecular geometry of *4*O-PPPyCN (calculated by MM2) and the plot of transition temperatures of heating and cooling cycles as a function of the terminal alkoxy chain of the *m*O-PPPyCN (*m* = 2–8) homologs.

Figure 3 shows the plot of transition temperatures of heating and cooling cycles against the number of carbon atoms in the terminal alkoxy chain for the *m*O-PPPyCN homologs [25]. In contrast to the purely nematic series of *n*O-PPPyMe homologs, all *m*O-PPPyCN (*m* = 2–8) compounds exhibited both enantiotropic nematic and smectic A phases. The longer alkoxy homologs (*m* = 7 and 8) exhibited even an additional enantiotropic smectic C phase. The occurrence of the smectic A phase manifests that strong intermolecular lateral forces help in forming a lamellar packing structure. In other words, in the series of *m*O-PPPyCN homologs, the strong polar cyanopyridine terminus aided the intermolecular polar head-to-head attraction, thus forming a dimer. The formation of the dimer not only elongated the longitudinal length of the bimolecular association but also enhanced the bimolecular lateral interaction force. Thus, the lamellar packing smectic A phase appeared.

When heated to higher temperatures, the vigorously turbulent motions of the alkoxy chains of *m*O-PPPyCN compounds became pronounced. At a certain characteristic temperature, *T_SmA-N_*, the lamellar packing structure of the smectic A phase collapsed, and the nematic phase appeared. The *T_NI_* of *m*O-PPPyCN (317, 314, 276.2, 269.3, 260.8, and 256.3 °C for *m* = 3–8, respectively) was considerably higher than those of *n*O-PPPyMe (232.8, 232.9, 220.9, 217.7, 209.9, and 206.9 °C for *n* = 3–8, respectively). The differences in *T_NI_* between the two series of *m*O-PPPyCN and *n*O-PPPyMe were considerably high (84.2, 81.1, 55.3, 51.6, 50.9, and 49.4 °C for alkyl group = 3–8, respectively) that the dimer associations are presumably existent in the fluid nematic phase of *m*O-PPPyCN compounds.

Compared with the nematic phase lengths of *n*O-PPPyMe (29.0, 35.9, 35.0, 42.7, 37.8, and 38.0 °C for *n* = 3–8, respectively), those of *m*O-PPPyCN at heating (69, 82, 64.5, 79.4, 21, and 6.3 °C for *m* = 3–8, respectively) were substantially wider when the alkoxy chain lengths were short (*m* = 3–6) but were severely reduced as the alkoxy chain lengthened (*m* = 7 and 8). Apparently, the nematic phase was favored in the series of *m*O-PPPyCN homologs when alkoxy chain lengths were short. However, the nematic phase lengths were shortened and replaced extensively by the appearance of smectic phases when *m* = 7 and 8. The geometrically asymmetric effect of the pyridine moiety that favored the nematic phase was apparently obscured when the alkoxy chain lengths were long.

As the alkoxy chain lengthened (*m* ≥ 7), the smectic phase was further reinforced. Not only was the smectic A phase length greatly extended but the smectic C phase appeared as well. In other words, the lamellar packing structures of *7*O-PPPyCN and *8*O-PPPyCN were further enhanced from the participation of lateral attractive forces of those lengthened alkoxy chains. Overall, the strongly polar cyanopyridine terminus in *m*O-PPPyCN homologs enhanced the occurrence of both nematic and smectic phases.

The freezing points of *m*O-PPPyCN (201.4, 184.1, 163.8, 150.8, 142.9, and 133.6 °C for *m* = 3–8, respectively) decreased as the alkoxy chains lengthened but varied in a relatively wide temperature range of 67.8 °C. By contrast, those of *n*O-PPPyMe (196.0, 186.9, 181.1, 171.8, 169.0, and 165.7 °C for *n* = 3–8, respectively) decreased as alkoxy chains lengthened, but varied only in a short temperature range of 30.3 °C. The wide variation of freezing points in the series of *m*O-PPPyCN homologs corroborates the evidence of existence of the dimer structure in the smectic phases of the homologs when solidification occurred. Furthermore, the geometrical resemblance of mesogenic cores between the two series of compounds made the freezing points of *4*O-PPPyCN and *4*O-PPPyMe appear at approximately the same temperature (184.1 and 186.9 °C, respectively), although the polarity of the terminus in these two series of compounds was quite different.

**Figure 4:**
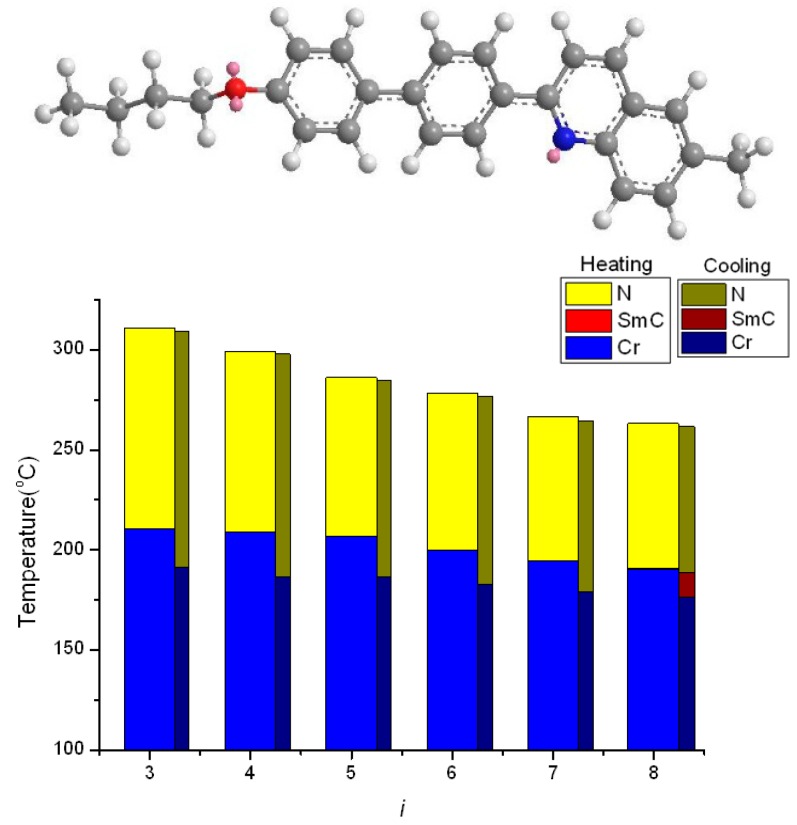
Molecular geometry of *4*O-PPQMe (calculated by MM2) and the plot of transition temperatures of heating and cooling cycles as a function of the terminal alkoxy chain of the *i*O-PPQMe (*i* = 3–8) homologs.

Figure 4 displays the plot of transition temperatures of heating and cooling cycles *versus* the number of carbon atoms in the terminal alkoxy chain of the *i*O-PPQMe homologs [34]. When pyridine in *n*O-PPPyMe was replaced by quinoline, the longitudinal molecular length was extended; moreover, a kinked terminus was formed in the *i*O-PPQMe molecule. The geometrically asymmetric picoline terminus in *n*O-PPPyMe led to the nematic phase solely; similarly, the geometrically kinked 6-methylquinoline terminus in *i*O-PPQMe led to the purely nematic phase, although *8*O-PPQMe exhibited an additional short-range of enantiotropic smectic C phase (0.7 and 12.5 °C, for heating and cooling cycles, respectively). Furthermore, the *T_NI_* and nematic phase lengths of *i*O-PPQMe homologs were considerably higher and longer than those of *n*O-PPPyMe homologs.

Exceptionally wide nematic phase lengths of the *i*O-PPQMe compounds (100, 90.1, 79.1, 78.1, 71.9, and 72.2 °C for *i* = 3–8, respectively) were observed on heating. Similar behaviors were observed during cooling. The wide nematic phase lengths can be ascribed to both the dimer formation, which substantially increased the *T_NI_* (or *T_IN_*), and the kinked methylquinoline terminus, which substantially reduced the melting and freezing transition temperatures. Because of the poly-aromatic nature of the *i*O-PPQMe compounds, these compounds were barely soluble in chloroform, and their ^13^C-NMR spectra could not be obtained.

The *T_NI_* of *i*O-PPQMe homologs (311, 299.1, 286.0, 278.1, 266.4, and 263.1 °C for *i* = 3–8, respectively, at heating) decreased almost monotonically as the alkoxy chains lengthened. The odd-even effect of *T_NI_* was not observed because the molecular width of *i*O-PPQMe was extended by the kinked quinoline moiety. Thus, an alkoxy chain in the series of *i*O-PPQMe compounds merely functioned as an impurity to its pseudo-crystalline nematic phase. In other words, the longer the alkoxy chain length, the lower the *T_NI_*.

The *T_NI_* of *i*O-PPQMe homologs was considerably higher than those of *n*O-PPPyMe homologs (232.8, 232.9, 220.9, 217.7, 209.9, and 206.9 °C for *n* = 3–8, respectively). The differences in the *T_NI_* between *i*O-PPQMe and *n*O-PPPyMe homologs were 78, 66.2, 65.1, 60.4, 56.5, and 56.2 °C for alkyl groups = 3–8, respectively. These differences are comparable with those between *m*O-PPPyCN and *n*O-PPPyMe (84.2, 81.1, 55.3, 51.6, 50.9, and 49.4 °C for alkyl groups = 3–8, respectively), thus, strongly indicating dimer formation in the fluid nematic phase of *i*O-PPQMe compounds. Dimer formation is possibly a result of the bimolecular head-to-head quinoline interaction of *i*O-PPQMe compounds.

Compared with the freezing points of nO-PPPyMe (196.0, 186.9, 181.1, 171.8, 169.0, and 165.7 °C for *n* = 3–8, respectively), which occurred in a range of 30.3 °C, those of *i*O-PPQMe (191.5, 186.7, 186.6, 182.7, 179.4, and 176.2 °C for *i* = 3–8, respectively) appeared in a narrow range of 15.3 °C. The freezing points of *i*O-PPQMe compounds were apparently dominated by the kinked quinoline moiety; thus, they decreased only slightly with increasing alkoxy chain lengths. On the other hand, the differences in freezing points between these two series of compounds are rather small even though an extra fused benzene ring was present in *i*O-PPQMe, thus, indicating the important role of the slightly distorted hexagon, pyridine, in dominating the freezing points.

**Figure 5:**
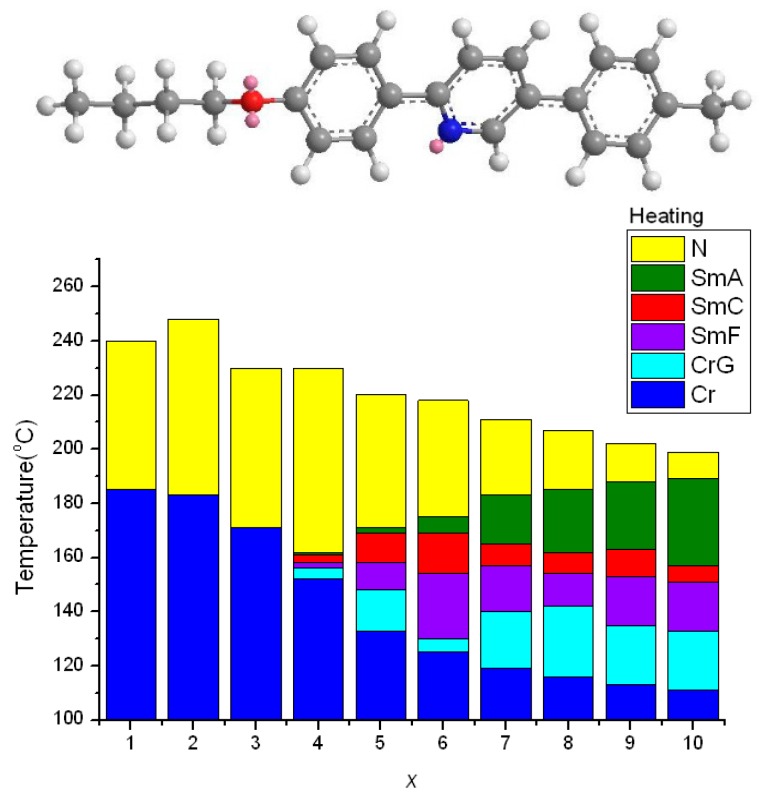
Molecular geometry of *4*O-PPyPMe (calculated by MM2) and the plot of transition temperatures of heating cycle as a function of the terminal alkoxy chain of the *x*O-PPyPMe (*x* = 1–10) homologs.

Figure 5 displays the plot of transition temperatures of the heating cycle against the number of carbon atoms in the alkoxy group in *x*O-PPyPMe compounds [14]. When pyridine in *n*O-PPPyMe was switched to the middle of the teraryl mesogenic core, *x*O-PPyPMe compounds were formed. When the geometrically asymmetric pyridine moiety was situated in the middle of the teraryl mesogenic core, bending position of the teraryl mesogenic core was there too. Thus, the overall bending shape of *x*O-PPyPMe compounds was more pronounced than that of *n*O-PPPyMe compounds. Consequently, the nematic phase was favored and was the only mesophase observed when alkoxy chain lengths were short (*x* = 1–3). Furthermore, the nematic phase length of *3*O-PPyPMe (230 °C − 171 °C = 59 °C) was much longer than that of *3*O-PPPyMe (232.8 °C − 203.8 °C = 29.0 °C) because of the low melting point of *3*O-PPyPMe compared with that of *3*O-PPPyMe. Nevertheless, the nematic phase of *x*O-PPyPMe homologs was observed not only when *x* = 1 but also persistently through the whole series to *x* = 10. These data illustrate the importance of bending or bending position of a mesogenic core on lowering the melting point and enhancing the occurrence of the nematic phase.

An odd–even effect of *T_NI_* was found in the series of *x*O-PPyPMe homologs when the alkoxy chain lengths were short (240, 248, and 230 °C for *x* = 1–3, respectively). Subsequently, the *T_NI_* decreased gradually (230, 220, 218, 211, 207, 202, and 199 °C for *x* = 4–10, respectively) as the alkoxy chains lengthened. A pseudo stepwise decrease in *T_NI_* was found in the series of *x*O-PPyPMe homologs when *x* = 3–8, if the *T_NI_* were grouped into three pairs: *x* = 3 and 4; 5 and 6; and 7 and 8. Mild damping in the stepwise decrease in the *T_NI_* was also observed.

The *T_NI_* of the series of *x*O-PPyPMe compounds (230, 230, 220, 218, 211, and 207 °C for *x* = 3–8, respectively) at heating were comparable to those of *n*O-PPPyMe compounds (232.8, 232.9, 220.9, 217.7, 209.9, and 206.9 °C for *n* = 3–8, respectively). If the *T_NI_* was considered as an indicator of nematic phase stability [35], the nematic phase stabilities of the two series of compounds (*x*O-PPyPMe and *n*O-PPPyMe) were nearly identical. In other words, nematic phase stability was independent of the bending position (the position of pyridine moiety) in the teraryl mesogenic core.

The nematic phase length on heating was wide and alternated in the beginning for the *x*O-PPyPMe series (55, 65, 59, 69, and 50 °C for *x* = 1–5, respectively) and then decreased monotonically (50, 44, 29, 23, 15, and 9 °C for *x* = 5–10, respectively) as the alkoxy chains lengthened. In comparison, the nematic phase length of *n*O-PPPyMe compounds were in a relatively constant range of 35.9 °C ± 6.9 °C (29.0, 35.9, 35.0, 42.7, 37.8, and 38.0 °C for *n* = 3–8, respectively). Apparently, the nematic phase was favored for the terminal pyridine moiety when the alkoxy chains were long and was favored for the middle pyridine moiety when alkoxy chains were short.

When pyridine was situated in the middle of the teraryl mesogenic core as in *x*O-PPyPMe compounds, the dipole of pyridine greatly enhanced the intermolecular lateral attractive force. Thus, smectic phases were favored. The disposition of the smectic phase of *x*O-PPyPMe compounds was further enhanced as the alkoxy chains lengthened. Therefore, polymorphism was observed when *x* ≥ 4. As *x* increases, the lengths of a plethora of smectic phases increase gradually, whereas the length of the nematic phase decreases. In comparison, no smectic phase was revealed in the series of *n*O-PPPyMe (*n* = 3–8) compounds. It was surprising how strongly a pyridine moiety can induce the occurrence of smectic phases when it is situated in the middle of a teraryl mesogenic core.

The melting points of *x*O-PPyPMe compounds (171, 152, 133, 125, 119, and 116 °C for *x* = 3–8, respectively) were considerably lower than those of *n*O-PPPyMe (203.8, 197.0, 185.9, 175.0, 172.1, and 168.9 °C for *n* = 3–8, respectively). This finding illustrates that the bending position of a teraryl mesogenic core can substantially influence the molecular packing in a solid crystalline phase. Consequently, the mesomorphic lengths (both nematic and smectic phases) of *x*O-PPyPMe (59, 78, 87, 93, 92, and 91 °C for *x* = 3–8, respectively) were much longer than those of *n*O-PPPyMe (29.0, 35.9, 35.0, 42.7, 37.8, and 38.0 °C for *n* = 3–8, respectively). Thus, a slight bend in the middle of a teraryl mesogenic core is useful to disrupt the linear symmetry of a LC molecule. This disruption not only reduces the melting points, but also enhances the early occurrence or appearance of the mesophase and even widens the mesomorphic length.

Most *p*-terphenyl LCs synthesized were symmetrical dialkyl or dialkoxy compounds, which showed either no mesophase or highly organized smectic phases. For example, dimethyl and diethyl *p*-terphenyls exhibited no mesophase and melted at 256 and 228 °C, respectively [36]. Unsymmetrical dialkyl or dialkoxy *p*-terphenyls were rarely synthesized and smectic phases were usually observed. For example, propyl, pentyl *p*-terphenyl has a sequence of smectic phases of E, B, A, from crystalline to isotropic phases at temperatures of 180, 200, 214, and 218 °C, respectively [37]. Even if a nematic phase appears, unsymmetrical *p*-terphenyls usually exhibit a nematic phase monotropically or at a high temperature range. For example, ethoxy, butoxy *p*-terphenyl not only exhibits smectic phases but also exhibits a short range of nematic phase at 270–280 °C [37]. By contrast, *1*O-PPyPMe has a pyridine in the middle of its teraryl ring and shows a wide range of nematic phase from 185 to 240 °C. How delicate and subtle the mesomorphic phases of LC molecule response to its molecular structure!

The effect of pyridine in this article can be summarized as follows:

- The geometrically asymmetric effect of pyridine moiety favors the formation of nematic phase when alkyl chain lengths are short, but the effect will be obscured when alkyl chain lengths are long and smectic phase appears.
- The distorted hexagonal pyridine moiety will reduce melting and freezing temperatures. The effect is pronounced when alkoxy chains are short and even, and is somewhat obscured when alkoxy chains are long.
- An enlarged effect of pyridine can be found using quinoline moiety, which substantially increase the *T_NI_* (or *T_IN_*) and reduce the melting and freezing transition temperature, thus providing a wide nematic phase length.
- When pyridine situated in the middle of the teraryl mesogenic core, the nematic phase is favored when alkyl chains are short, and nematic phase length is enlarged because of reduced melting point, however, smectic phases are favored, and polymorphism will be observed when alkyl chains are long.

## 3. Experimental Section

### 3.1. General

Chemical structures of these targeted compounds were characterized by ^1^H and ^13^C-NMR spectra using a Bruker AC 300 spectrometer (Bruker Corporation, Billerica, MA, USA). Infrared spectra were obtained on a Perkin-Elmer 1600 Series spectrometer (Perkin-Elmer, Norwalk, CT, USA). The purity of these compounds was monitored by thin-layer chromatography and reconfirmed by elemental analysis.

Mesomorphic phases were mainly characterized from microscopic texture of samples sandwiched between two glass plates under a polarizing optical microscope (POM; Olympus BH-2, Two Corporate Center Drive, Melville, NY, USA) equipped with a Mettler FP90/FP82HT hot stage (Mettler, Columbus, OH, USA). Phase transition temperatures and their corresponding transition enthalpies were determined by differential scanning calorimetry (DSC), using a Perkin-Elmer DSC 7 calorimeter at a scanning rate of 5 °C·min^−1^. Heating and cooling cycles were repeated. Only those data from the first cooling and second heating cycles are reported.

### 3.2. Synthesis

4-Bromo-4’-hydroxybiphenyl was purchased from Aldrich Chemical Co. (Saint Louis, MO, USA) and used as-received. Phenyl chloroformate, 3-methylpyridine and *n*-bromoalkanes were distilled under inert atmosphere immediately before use. Anhydrous toluene and tetrahydrofuran (THF) were refluxed over sodium and distilled before use. Column chromatography was done using silica gel (MN Kieselgel 60, 70–230 mesh; Duren, Germany). The purity of the compounds was monitered by thin-layer chromatography and reconfirmed by elemental analysis. The synthetic method of 2-(4-alkoxybiphenylen-4′-yl)-5-methylpyridines was outlined in Scheme 1.

#### Representative Procedure for Homologs of 2-(4-Alkoxybiphen-4′-yl)-5-methylpyridines (*n*O-PPPyMe, *n* = 3–8)

A short 2-step process with overall yields in the range of 34%–38% was obtained (Table 1). For *4*O-PPPyMe in **3**, first, freshly dried magnesium granules (11 mmol) were added to a solution of 4-bromo-4’-butoxybiphenyl (10 mmol) in THF (20 mL) to form Grignard solution **1** under an inert atmosphere for about 0.5 h. Then, the Grignard solution **1** was slowly added by a syringe into a preformed solution of 3-methylpyridinium chloride **2**, which was prepared from phenyl chloroformate (10 mmol) and 3-methylpyridine (10 mmol) in dry THF (20 mL) at −20 °C for 0.5 h. The resulting solution was left to warm up slowly to room temperature and stirred for another 8 h. After evaporation of the solvent THF, the residue was extracted with Et_2_O. The organic layer was washed, once with 20% NH_4_Cl solution and twice with distilled water and brine, and dried with magnesium sulfate. For *4*O-PPPyMe in **4**, about 1.1 eq. *o*-chloranil was added to a solution of dry toluene (20 mL) and compound **3** (10 mmol). The reaction mixture was refluxed under inert atmosphere for about 3 h and then quenched by addition of 1 N NaOH (25 mL) and Et_2_O (25 mL) and filtration through Celite (Duren, Germany). After normal aqueous work-up and isolation with column chromatography (methylene chloride: ethyl acetate = 30:1), an overall 2-step reaction with fair yield of 2-(4-butoxybiphen-4′-yl)-5-methylpyridine **4** (37%) was afforded. The crude oxidized product of **4** was further purified by re-crystallisation several times from a mixed solvent of methylene chloride and ethyl acetate (4:1). The other *n*O-PPPyMe homologues were synthesized essentially by the same procedure as described above for the *n* = 4 homologue (Table 1). Satisfactory ^1^H-NMR, ^13^C-NMR, IR and elemental analysis results were obtained for all compounds as listed below.

*2-(4-Propoxybiphen-4′-yl)-5-methylpyridine (3O-PPPyMe).* ^1^H-NMR (CDCl_3_): δ 8.53 (s, 1 H, pyridine), 8.02 (d, 2 H, *J* = 8.4 Hz, center phenyl near pyridine), 7.54–7.68 (m, 6 H, 4 in phenyl, 2 in pyridine), 6.99 (d, 2 H, *J* = 8.7 Hz, outer phenyl near oxygen), 3.98 (t, 2 H, *J* = 6.6 Hz, -CH_2_), 2.38 (s, 3 H, -CH_3_ in pyridine), 1.84 (sext, 2 H, *J* = 6.9 Hz, -CH_2_), 1.06 (t, 3 H, *J* = 7.5 Hz, -CH_3_). ^13^C-NMR (CDCl_3_): ppm 159.1, 154.7, 150.3, 141.2, 137.8, 137.5, 133.2, 131.7, 128.2, 127.2, 127.1, 120.0, 115.1, 69.8, 22.8, 18.4, 10.7. IR (ATR): cm^−1^ 3038 (aromatic C–H stretch), 3009 (aromatic C–H stretch), 2960 (aliphatic C–H stretch), 2931 (aliphatic C–H stretch), 2875 (aliphatic C–H stretch), 1602 (benzene ring stretch), 1499 (benzene ring stretch), 1474 (pyridine ring stretch), 1393 (pyridine ring stretch), 1377 (pyridine ring stretch), 1249 (asymmetric C–O–C stretch), 1199 (symmetric C–O–C stretch), 975 (out-of-plane C–H bend), 817 (out-of-plane C–H bend). Anal. calcd for C_21_H_21_NO: C, 83.13; H, 6.98; N, 4.62. Found: C, 82.89; H, 6.94; N, 4.61.

*2-(4-Butoxybiphen-4′-yl)-5-methylpyridine (4O-PPPyMe).* ^1^H-NMR (CDCl_3_): δ 8.53 (dd, 1 H, *J*_1_ = 1.5 Hz, *J*_2_ = 0.6 Hz, pyridine), 8.03 (d, 2 H, *J* = 8.4 Hz, center phenyl near pyridine), 7.54–7.68 (m, 6 H, 4 in phenyl, 2 in pyridine), 6.99 (d, 2 H, *J* = 8.7 Hz, outer phenyl near oxygen), 4.02 (t, 2 H, *J* = 6.6 Hz, -CH_2_), 2.38 (s, 3 H, -CH_3_ in pyridine), 1.80 (quin, 2 H, *J* = 6.9 Hz, -CH_2_), 1.52 (sext, 2 H, *J* = 7.5 Hz, -CH_2_), 1.00 (t, 3 H, *J* = 7.5 Hz, -CH_3_). ^13^C-NMR (CDCl_3_): ppm 159.1, 154.6, 150.3, 141.2, 137.8, 137.5, 133.1, 131.7, 128.2, 127.2, 127.1, 120.0, 115.0, 68.0, 31.5, 19.4, 18.4, 14.1. IR (ATR): cm^−1^ 3038 (aromatic C–H stretch), 3009 (aromatic C–H stretch), 2956 (aliphatic C–H stretch), 2933 (aliphatic C–H stretch), 2871 (aliphatic C–H stretch), 1601 (benzene ring stretch), 1499 (benzene ring stretch), 1466 (pyridine ring stretch), 1395 (pyridine ring stretch), 1377 (pyridine ring stretch), 1248 (asymmetric C–O–C stretch), 1198 (symmetric C–O–C stretch), 968 (out-of-plane C–H bend), 817 (out-of-plane C–H bend). Anal. calcd for C_22_H_23_NO: C, 83.24; H, 7.30; N, 4.41. Found: C, 82.95; H, 7.30; N, 4.39.

*2-(4-Pentoxybiphen-4′-yl)-5-methylpyridine (5O-PPPyMe).* ^1^H-NMR (CDCl_3_): δ 8.53 (dd, 1 H, *J*_1_ = 1.5 Hz, *J*_2_ = 0.6 Hz, pyridine), 8.03 (d, 2 H, *J* = 8.4 Hz, center phenyl near pyridine), 7.54–7.68 (m, 6 H, 4 in phenyl, 2 in pyridine), 6.99 (d, 2 H, *J* = 8.7 Hz, outer phenyl near oxygen), 4.01 (t, 2 H, *J* = 6.6 Hz, -CH_2_), 2.38 (s, 3 H, -CH_3_ in pyridine), 1.82 (quin, 2 H, *J* = 6.9 Hz,-CH_2_), 1.36–1.52 (m, 4 H, -CH_2_), 0.95 (t, 3 H, *J* = 7.2 Hz, -CH_3_). ^13^C-NMR (CDCl_3_): ppm 159.1, 154.6, 150.3, 141.2, 137.8, 137.5, 133.1, 131.7, 128.2, 127.2, 127.1, 120.0, 115.0, 68.3, 29.2, 28.4, 22.7, 18.4, 14.2. IR (ATR): cm^−1^ 3038 (aromatic C–H stretch), 3008 (aromatic C–H stretch), 2959 (aliphatic C–H stretch), 2932 (aliphatic C–H stretch), 2871 (aliphatic C–H stretch), 1601 (benzene ring stretch), 1498 (benzene ring stretch), 1466 (pyridine ring stretch), 1395 (pyridine ring stretch), 1376 (pyridine ring stretch), 1248 (asymmetric C–O–C stretch), 1195 (symmetric C–O–C stretch), 986 (out-of-plane C–H bend), 818 (out-of-plane C–H bend). Anal. calcd for C_23_H_25_NO: C, 83.34; H, 7.60; N, 4.23. Found: C, 82.98; H, 7.56; N, 4.20.

*2-(4-Hexoxybiphen-4′-yl)-5-methylpyridine (6O-PPPyMe).* ^1^H-NMR (CDCl_3_): δ 8.53 (dd, 1 H, *J*_1_ = 1.5 Hz, *J*_2_ = 0.6 Hz, pyridine), 8.02 (d, 2 H, *J* = 8.4 Hz, center phenyl near pyridine), 7.55–7.68 (m, 6 H, 4 in phenyl, 2 in pyridine), 6.98 (d, 2 H, *J* = 8.7 Hz, outer phenyl near oxygen), 4.01 (t, 2 H, *J* = 6.6 Hz, -CH_2_), 2.38 (s, 3 H, -CH_3_ in pyridine), 1.81 (quin, 2 H, *J* = 6.6 Hz, -CH_2_), 1.44–1.53 (m, 2 H, -CH_2_), 1.33–1.42 (m, 4 H, -CH_2_), 0.92 (t, 3 H, *J* = 7.2 Hz, -CH_3_). ^13^C-NMR (CDCl_3_): 159.1, 154.6, 150.3, 141.2, 137.8, 137.5, 133.1, 131.7, 128.2, 127.2, 127.1, 120.0, 115.0, 68.3, 31.8, 29.4, 25.9, 22.8, 18.4, 14.2. IR (ATR): cm^−1^ 3040 (aromatic C–H stretch), 2990 (aromatic C–H stretch), 2952 (aliphatic C–H stretch), 2931 (aliphatic C–H stretch), 2870 (aliphatic C–H stretch), 1597 (benzene ring stretch), 1499 (benzene ring stretch), 1469 (pyridine ring stretch), 1393 (pyridine ring stretch), 1377 (pyridine ring stretch), 1249 (asymmetric C–O–C stretch), 1199 (symmetric C–O–C stretch), 1029 (out-of-plane C–H bend), 817 (out-of-plane C–H bend). Anal. calcd for C_24_H_27_NO: C, 83.44; H, 7.88; N, 4.05. Found: C, 82.76; H, 7.88; N, 4.04.

*2-(4-Heptoxybiphen-4′-yl)-5-methylpyridine (7O-PPPyMe).* ^1^H-NMR (CDCl_3_): δ 8.53 (dd, 1 H, *J*_1_ = 1.5 Hz, *J*_2_ = 0.6 Hz, pyridine), 8.03 (d, 2 H, *J* = 8.4 Hz, center phenyl near pyridine), 7.54–7.68 (m, 6 H, 4 in phenyl, 2 in pyridine), 6.99 (d, 2 H, *J* = 6.9 Hz, outer phenyl near oxygen), 4.01 (t, 2 H, *J* = 6.6 Hz, -CH_2_), 2.38 (s, 3 H, -CH_3_ in pyridine), 1.81 (quin, 2 H, *J* = 6.6 Hz, -CH_2_), 1.42–1.51 (m, 2 H, -CH_2_), 1.30–1.40 (m, 6 H, -CH_2_), 0.91 (t, 3 H, *J* = 6.6 Hz, -CH_3_). ^13^C-NMR (CDCl_3_): 159.1, 154.6, 150.3, 141.2, 137.8, 137.5, 133.1, 131.7, 128.2, 127.2, 127.1, 120.0, 115.0, 68.3, 32.0, 29.5, 29.3, 26.2, 22.8, 18.4, 14.3. IR (ATR): cm^−1^ 3039 (aromatic C–H stretch), 2988 (aromatic C–H stretch), 2957 (aliphatic C–H stretch), 2934 (aliphatic C–H stretch), 2859 (aliphatic C–H stretch), 1596 (benzene ring stretch), 1499 (benzene ring stretch), 1467 (pyridine ring stretch), 1394 (pyridine ring stretch), 1377 (pyridine ring stretch), 1249 (asymmetric C–O–C stretch), 1198 (symmetric C–O–C stretch), 1023 (out-of-plane C–H bend), 818 (out-of-plane C–H bend). Anal. calcd for C_25_H_29_NO: C, 83.52; H, 8.13; N, 3.90. Found: C, 82.81; H, 8.05; N, 4.20.

*2-(4-Octoxybiphen-4′-yl)-5-methylpyridine (8O-PPPyMe).* ^1^H-NMR (CDCl_3_): δ 8.53 (d, 1 H, *J* = 2.1 Hz, pyridine), 8.02 (d, 2 H, *J* = 6.6 Hz, center phenyl near pyridine), 7.55–7.68 (m, 6 H, 4 in phenyl, 2 in pyridine), 6.98 (d, 2 H, *J* = 8.7 Hz, outer phenyl near oxygen), 4.01 (t, 2 H, *J* = 6.6 Hz, -CH_2_), 2.38 (s, 3 H, -CH_3_ in pyridine), 1.81 (quin, 2 H, *J* = 6.6 Hz, -CH_2_), 1.43–1.50 (m, 2 H, -CH_2_), 1.25–1.35 (m, 8 H, -CH_2_), 0.89 (t, 3 H, *J* = 6.6 Hz, -CH_3_). ^13^C-NMR (CDCl_3_): 159.1, 154.7, 150.3, 141.2, 137.8, 137.5, 133.1, 131.7, 128.2, 127.2, 127.1, 120.0, 115.0, 68.3, 32.0, 29.6, 29.5, 29.4, 26.3, 22.9, 18.4, 14.3. IR (ATR): cm^−1^ 3040 (aromatic C–H stretch), 2988 (aromatic C–H stretch), 2956 (aliphatic C–H stretch), 2922 (aliphatic C–H stretch), 2858 (aliphatic C–H stretch), 1602 (benzene ring stretch), 1500 (benzene ring stretch), 1473 (pyridine ring stretch), 1394 (pyridine ring stretch), 1378 (pyridine ring stretch), 1250 (asymmetric C–O–C stretch), 1198 (symmetric C–O–C stretch), 1025 (out-of-plane C–H bend), 819 (out-of-plane C–H bend). Anal. calcd for C_26_H_31_NO: C, 83.52; H, 8.13; N, 3.90. Found: C, 83.52; H, 8.32; N, 3.95.

## 4. Conclusions

We present a convenient, short two-step synthesis method of a novel series of teraryl LC compounds, 2-(4-alkoxybiphen-4′-yl)-5-methylpyridines (*n*O-PPPyMe, *n* = 3–8). Through this two-step process, overall yields of 34%–38% were obtained. Short-step and fair-yield synthesis allowed us to readily access these compounds and study further their thermotropic properties.

Nematic phase was the only mesophase in the series of *n*O-PPPyMe (*n* = 3–8) compounds. In addition, the odd–even effect of *T*_NI_(*T*_IN_), nematic phase length, and melting and freezing points of *n*O-PPPyMe (*n* = 3–8) compounds were discussed. The mesomorphic behaviours between the target molecules, *n*O-PPPyMe (*n* = 3–8) and those of three compounds series, *m*O-PPPyCN (*m* = 2–8), *i*O-PPQMe (*i* = 3–8), and *x*O-PPyPMe (*x* = 1–10) were compared. Thus, the polar cyano and nonpolar methyl end groups, aspect ratio of the mesogenic core, and position of the pyridine moiety in the teraryl mesogenic core were compared and discussed. Consequently, the effect of pyridine on the mesophase of a teraryl LC molecule was demonstrated.

In conclusion, crucial information regarding the molecular structure and geometry containing the pyridine moiety that favors to produce a nematic phase was provided. For example, the occurrence of the nematic phase and nematic phase length can be enhanced, by a slightly bending mesogenic moiety, 2,5-pyridine, or a kinked mesogenic moiety, 2,6-quinoline. In addition, the nematic phase usually appears when the alkyl chains are short. The vigorously turbulent motions of these short alkoxy chains increased the intermolecular distance and impeded the formation of a lamellar structure. Nematic stability can be further increased by a mesogen with a polar terminus when head-to-head dimerization is easily formed. Furthermore, an odd–even effect of nematic phase length and *T*_NI_ (or *T*_IN_) was usually observed because of both greater anisotropy of molecular polarizability (thus raising *T_NI_*) and stronger influence of an asymmetric mesogenic core on reducing melting and freezing temperatures for the alkoxy chains with an even number of carbons.

## Figures and Tables

**Table 1:** Yields of 2-(4-alkoxybiphen-4′-yl)-5-methylpyridines (*n*O-PPPyMe, *n* = 3–8).

Entry (*n*)	Alkyl Group	Yield ^a^ (%)
3	Propyl	36
4	Butyl	37
5	Pentyl	34
6	Hexyl	36
7	Heptyl	35
8	Octyl	38

^a^ Isolated yields are quoted after re-crystallization.

**Table 2:** Phase transition temperatures (°C) and the corresponding transition enthalpies (kJ·mol^−1^), in parentheses, for the homologous series of *n*O-PPPyMe, *n* = 3–8, compounds.

Compound *n*O-PPPyMe (*n*)	Phase Transition Temperatures (°C) and Their Corresponding Transition Enthalpies (kJ·mol^−1^)	
	Heating	Cooling
3	Cr 203.8(21.41) N 232.8(0.79) I	I 231.2(0.74) N 196.0(21.10) Cr
4	Cr 197.0(20.89) N 232.9(0.78) I	I 231.2(0.87) N 186.9(20.55) Cr
5	Cr 185.9(19.07) N 220.9(0.95) I	I 219.1(1.00) N 181.1(18.58) Cr
6	Cr 175.0(10.97) N 217.6(0.67) I	I 216.0(0.59) N 171.8(9.70) Cr
7	Cr 172.1(10.95) N 209.9(0.59) I	I 208.0(0.55) N 169.0(9.62) Cr
8	Cr 168.9(10.25) N 206.9(0.80) I	I 205.2(0.78) N 165.7(10.10) Cr

Scan rate: 5 °C·min^−1^; Cr = crystal phase, N = nematic phase, I = isotropic phase.
